# Do MZ twins have discordant experiences of friendship? A qualitative hypothesis-generating MZ twin differences study

**DOI:** 10.1371/journal.pone.0180521

**Published:** 2017-07-20

**Authors:** Kathryn Asbury, Nicola Moran, Robert Plomin

**Affiliations:** 1 Psychology in Education Research Centre, Department of Education, University of York, York, United Kingdom; 2 Department of Social Policy and Social Work, University of York, York, United Kingdom; 3 Social, Genetic and Developmental Psychiatry Centre, Institute of Psychiatry, King’s College London, London, United Kingdom; Istituto Superiore Di Sanita, ITALY

## Abstract

Using a qualitative monozygotic (MZ) twin differences design we explored whether adolescent MZ twins report discordant peer relationships and, if so, whether they perceive them as causes, consequences or correlates of discordant behaviour. We gathered free-response questionnaire data from 497 families and conducted in-depth telephone interviews with 97 of them. Within this dataset n = 112 families (23% of the sample) described discordant peer relationships. Six categories of discordance were identified (peer victimisation, peer rejection, fewer friends, different friends, different attitudes to friendship and dependence on co-twin). Participants described peer relationship discordance arising as a result of chance occurrences, enhanced vulnerability in one twin or discordant behaviour. Consequences of discordant peer relationships were seen as discordance in self-confidence, future plans, social isolation, mental health and interests. In all cases the twin with worse peer experiences was seen as having a worse outcome. Specific hypotheses are presented.

## Introduction

Behavioural genetic studies have confirmed that there are both genetic and environmental influences on human behaviour [1]. In the majority of cases the most influential environments are individual-specific, or non-shared, making us differ from those we are raised with [2–4]. However, non-shared environment (NSE), while recognised as a major source of behavioural variation, remains poorly understood and under-explored. This manuscript reports one strand of an unprecedentedly large qualitative monozygotic (MZ) twin differences study which was designed to address this dearth of understanding by taking an inductive approach to generating new, testable hypotheses about NSE [5]. We present findings related to peer relationships as one potential aspect of NSE.

Back in 1998 Judith Rich Harris made a case that peers are the primary agents of socialisation and development, and argued that we should look to peer relationships as the most likely tangible explanation of non-shared variation in personality and behaviour [6]. Exploring whether MZ twins have different experiences of peer relationships, and whether they perceive peer-relationship discordance as related to discordant behaviour, partially addresses this hypothesis. Differences between MZ twins have to be explained by NSE because MZ twins share their genes and much of their upbringing. An MZ differences design, based on within-pair discordance, can therefore hold constant the effects of genes and many aspects of the family environment, making it possible to develop hypotheses about environmentally mediated relationships between experiences and behaviour.

Identifying specific NSE experiences that can explain large proportions of phenotypic variance has been an unsuccessful endeavour, just as identifying single genes with large effects has proven a fruitless, and now abandoned, line of inquiry [7–9]. While specific NSE factors have certainly been identified they, like specific genes, tend to explain only a very small proportion of variance [7]. This consistent pattern has given rise to a hypothesis that NSE variance is best explained by chance–by unpredictable, transient experiences that affect individuals but do not generalise to groups [7]. This hypothesis is firmly rooted in empirical data and remains a genuine possibility, although it has been described as “a gloomy prospect” [3]. A case can still be made that small effects might accumulate to have large outcomes [10, 4]. It also remains true that we consistently find evidence of measured NSE that can explain variance in behaviour–just not very much of it, typically 1–5% [e.g. 11,12].

Two further hypotheses (other than all NSE variance being explained by chance) have emerged in the literature: [1] that measurable NSE experiences are most likely to have causal effects such that differences in experience will explain differences in behaviour [3,4]; and [2] that apparently NSE experiences are most likely to be the outcome of selection effects such that differences in behaviour will explain differences in experience [12–14].

Judith Rich Harris’ thesis in *The Nurture Assumption* [6] met with a substantial backlash [15,16]. However, criticism was not targeted at her argument that peers are important, but rather at her argument that parents aren’t. Harris was accused, with some justification, of throwing the baby out with the bathwater. However, the peers hypothesis was accepted without demur, most likely because it was a good fit with people’s intuitions and experience as well as with empirical evidence. In addition to behavioural genetic evidence pointing to the substantial importance of the NSE there is a large body of research that suggests the importance of peers to healthy development, particularly in adolescence–a time when exposure to peers is often very high [17,18]. What is surprising is that Harris’ hypothesis that peer relationships should explain a substantial proportion of NSE variance has not been subjected to a great deal of empirical testing.

That said, there has been some good research in this area and studies have yielded support for peers as an agent of NSE or, at least, a genuinely environmental variable. For instance, several studies have found variation in aspects of peer relationships to be primarily non-shared in origin. In one study which used two independent samples–one of adoptive and non-adoptive siblings and another of mixed sibling types (including twins)– 70–80% of the total variance in self-reported peer group delinquency was explained by NSE effects [19]. These findings were later replicated with teacher- and observer-report data, offering strong empirical support for Harris’ theory that peer relationships represent a truly environmental influence [20]. The same study also found peer group popularity to be substantially explained by NSE factors, albeit with some genetic influence [19]. Peer group college orientation, however, was found to be moderately heritable, with approximately half of the variance explained by genetic factors–a finding also reported elsewhere [21].

It should be noted that Manke et al. also found parent-reported peer group delinquency and popularity to be moderately to strongly heritable. Other studies have observed the same pattern of small to moderate heritability for peer group delinquency [22–25]. Manke et al. [21] also used a ‘best friends’ measure in which positive and negative dimensions of friendship were defined. The researchers found the positive dimension to be moderately heritable (h^2^ = .31) but the negative dimension to be primarily explained by NSE effects. Other studies have noted evidence of genotype correlation as an explanation of, for instance, the association between peer victimization and physical ill health [26] and the association between peer aggression and aggressive behaviour [27]. In summary, the picture is somewhat unclear but it is true to say that all studies find NSE factors to explain variation in peer relationships. The differences between the studies are of degree, and of whether significant genetic effects are also observed.

Studies have found that discordant friendships in adolescence can account for NSE variance in externalising behaviour [28,29], aspirations [30] and adult self-reported life satisfaction and relationship quality [31], lending some support to the causation hypothesis. Most recently, discordant peer victimization was found to account for NSE variation in daily cortisol secretions, along with discordance in the mother-child relationship [32]. However, most of these studies–not including Marion et al. [31]–have tended to rely on cross-sectional correlational designs in which the direction of effects remains unclear. It has therefore been convincingly argued that assumptions of causality–of NSE influence rather than NSE selection–are premature because the direction of causation could be in either or both directions [12]. However, a recent longitudinal study presented findings which indicate that being bullied is predictive of mental illness and, using an MZ differences model, found that the association was mediated environmentally [33]. This suggests that very severe peer relationship problems may act as genuinely environmental influences on mental health outcomes.

The vast majority of research in this area has focused on the relationship between antisocial behaviour and deviant peer affiliation–the ‘wrong crowd’ hypothesis [28,12]. By contrast, in this more developed area of genetically-informed peer research, support for the NSE ‘selection’ hypothesis has been clear. For instance, Burt and colleagues [12] used a longitudinal cross-lagged MZ differences design to look at the relationship between externalising behaviour and deviant peer affiliation at ages 14 and 17. The study found moderate to strong cross-sectional associations but, longitudinally, it showed that MZ discordance in externalising behaviour at age 14 predicted MZ discordance in deviant peer affiliation at age 17, but not the other way around. The finding was consistent with an earlier study [13] and provides strong support for the selection hypothesis. It appears, from studies such as these, that an identical twin displaying higher levels of externalising behaviour at one time point is more likely to have chosen or shaped worse behaved peers, relative to their co-twin, at a second time point. However, it is important to note that this still leaves the discordant externalising behaviour at the first time point to be explained by NSE factors. The focus on deviant peer affiliation as a candidate NSE factor has led to some imbalance in the field as it represents just one aspect of peer relationships, albeit an important one. A full typology of peer relationships is needed and could be useful to researchers attempting to map the non-shared environment. Peer relationship discordance in MZ twins is particularly notable as MZ twins have been found both in early childhood [34] and adolescence [35] to share more of their friends with one another than DZ twins [36,37].

The current study represents one strand of a larger qualitative hypothesis-generating MZ twin differences study in which adolescent MZ twins (and a parent) were asked to describe and explain differences between them in academic achievement, plans for the future and their lives and experiences more generally. We did not ask participants directly about peer relationships because a primary purpose of the study was for families to tell us their theories of discordance spontaneously. Instead, we waited to see whether, in line with Judith Rich Harris’ 1998 claim:

1. families would describe discordant peer relationships and, if so,
2. whether they would interpret them as causes (causal hypothesis), consequences (selection hypothesis) or simply correlates of discordant behaviour.

## Materials and methods

This study was approved by the Institute of Psychiatry Ethics Committee (PNM/11/12-142).

### Participants

We recruited a sub-sample of the UK Twins’ Early Development Study (TEDS), a longitudinal study of twins born in the UK between 1994 and 1996 [38]. Participants were recruited for this study in October 2012 and questionnaire data were gathered between October and December 2012. Discordant pairs were then identified for follow-up interviews which were conducted between February 2013 and February 2014. The TEDS sample has been found to be reasonably representative of the UK population of same-age adolescents and their parents [39]. For the current qualitative study 2,162 TEDS families with MZ twins were invited to take part and, of those, we received data from 497, a response rate of 23%. This was lower than hoped, which may reflect sample selectivity. The relatively increased proportion of girls in the current sample (from c.50% at first contact to 61%) is representative of TEDS at 16, although not of wider UK society. This significant discrepancy may be the result of greater willingness to engage with data collection among girls than boys at this age and stage. The current sample was also significantly higher in terms of SES (*M =* 0.31, compared to 0.00 at first contact and 0.1 at age 16) and *g* (general cognitive ability: measured at age 12; *M =* 0.11, compared to 0.00). All group mean differences were assessed with t-tests. TEDS families have been studied throughout their lives but this was the first occasion on which we had asked a sample of them to provide free-response data. There are indications that the approach was off-putting to some, potentially leading to a slightly biased sample. Although this does not matter in one sense, because our interest was in within-pair not between-family differences, it is important to bear the evidence of sample selectivity in mind. It remains possible that NSE influences are different for families in different circumstances.

Free-response questionnaire data were gathered from the n = 497 participating families with identical twins (61% female). Zygosity was confirmed using DNA for 84% (questionnaire data) and 85% (interview data) of participants. In the remaining cases zygosity was assigned via a questionnaire that has been found to be 95% accurate in the TEDS sample [40].

Three questionnaires were posted to each family and, in most cases, we received self-report data from a parent (usually mother) and both twins. The twins’ average age was 17.3 (range 16.2–18.9). After analysis of the questionnaires, telephone interviews were conducted with 97 families (both twins and one parent in most cases) who were selected because the twins reportedly showed strong signs of discordance in one or more aspects of achievement, behaviour or experience, suggesting NSE influence. In the course of the interviews and questionnaires n = 112 families spontaneously mentioned discordant experiences of peer relationships and these 112 families are the subject of the current study. To clarify, the sample included pairs who were not invited to take part in a telephone interview as well as those that were. Families were included in the current study if they spontaneously referred to discordance in peer relationships in either their questionnaire responses or during a telephone interview. Peer-discordance was usually described spontaneously in relation to another area of discordance, rather than in response to a direct question.

### Measures

New measures were developed for the current study and, other than information regarding zygosity and gender, existing TEDS data were not used. We took an inductive approach that was not rooted in previously gathered data. A 5-item screening questionnaire was designed to identify potential sources of discordance between identical twins towards the end of compulsory education. The first item asked whether twins performed differently in their General Certificates of Secondary Education (GCSEs) overall and, if so, what the differences were and how they might be explained. GCSEs are the public examinations taken by most UK students at the end of the academic year in which they turn 16. Most students take GCSEs in a broad range of academic subjects typically including English, Maths, Science, Humanities, Arts and, often, Languages. The second item focused on discordance in core GCSE subjects–English, Maths and Science–and asked whether there was a difference of at least two grades (e.g. A*/B or D/F) and how such discordance might be explained. The third question asked about discordance in next steps after GCSEs, namely whether students planned to pursue traditional academic qualifications (A Levels), vocational qualifications or work-based opportunities such as apprenticeships. The fourth item focused on discordance in hopes for the future and the fifth was a catch-all item: *What are the major differences (not already described) that you notice between Twin 1 and Twin 2*, *and how do you explain these differences*? Before sending the questionnaire to study participants we conducted a feasibility test with a small convenience sample of sixteen year olds in order to ensure that the items were suitable and clear for the age group. Small changes were made on the basis of this feasibility study. Data for the current study were drawn from answers to all items; that is, we noted evidence and discussion of peer discordance wherever it was spontaneously mentioned by twins or their parents. All items were open-ended as the aim was to ask families for their hypotheses about perceived discordance in a way that would not be leading.

Telephone interviews with twins and their parents were conducted by two experienced interviewers. Because of the hypothesis-generating nature of this study bespoke interview guides were drawn up by the researchers for each participant, focusing on the differences and explanations identified in the questionnaire. Researchers read the completed free-response questionnaires provided by each family selected for interview on the grounds of discordance (in a range of behaviours and experiences). They then documented all reasons offered by each member of the family to explain this discordance and turned the explanations into questions followed by a series of relevant probes. This formed a semi-structured interview schedule that differed by family. Also, when potential hypotheses were suggested in the interviews that had not been mentioned previously, interviewers probed for a full account of each participant’s view. This flexible approach was taken so that participants could give a full account of their beliefs about why one twin differed from the other, unrestricted by closed or standardised questions. Evidence and discussion of discordant experiences of friendship was documented as it arose.

### Procedure

Families invited to participate in the study received an information letter, consent form and three questionnaires–one for a parent and two for the twins. Separate envelopes for each participant were included so that individuals would be able to keep their responses private. Families returning completed sets of questionnaires received a £15 voucher. On receipt, questionnaire data were transcribed and entered into Excel.

Analysis of questionnaire data served two related purposes: (i) to indicate areas of discordance and possible explanatory factors for discordance between identical twins; and (ii) to aid selection of a sub-sample of families to be contacted for follow-up interviews.

Families selected for interview were contacted by telephone and asked for consent to participate. Times were then arranged to interview all three family members participating in the study. In cases where all family members were interviewed during the same telephone call they were asked not to be in the same room to ensure individual privacy. All interviews were recorded and transcribed with the full consent of participants.

### Analysis

All questionnaires and interview transcripts were initially coded by one researcher for evidence of within-pair discordance in peer relationships. In order to establish the reliability of coding, approximately 10% (50/497) of the questionnaires and 15% (15/97) of the interviews were then coded independently by a second researcher. There was a good degree of congruence (88% for questionnaires and 87% for interviews).

A more fine-grained approach to coding was then taken to the 112 families (23% of the full sample) who had described within-pair peer discordance (85 in their questionnaires; 11 in interviews; and 16 in both). Full data for each of these families was charted using the Framework approach [41] to order and synthesise the data through five stages: familiarisation; identifying conceptual themes; indexing; charting; and mapping. The Framework approach allows the sequential organisation and interpretation of qualitative data. A table is created which displays cases in rows, and themes or categories in columns. Taken together the rows and columns suggest explanations. The primary column in this analysis related to the type of discordance described and six categories of discordance were identified. In order to check inter-rater reliability a second researcher independently coded 10% of the dataset into the six types of peer-relationship discordance, and 92% congruence was achieved between raters. Small disagreements were discussed and minor adjustments made to the coding framework. The other columns in the Framework related to perceived causes and perceived consequences of the reported peer-relationship discordance.

MZ differences in experiences of friendship were then analysed in detail using each of the Framework’s categories to generate specific hypotheses about what MZ discordance in peer relationships looks like in this sample (a proposed typology); and what participants saw as the causes and consequences of the observed discordance. Interpretations and potential hypotheses were checked against the raw data and verified via on-going discussions between researchers.

## Results

Six categories of peer-relationship discordance were identified in questionnaire and/or interview data gathered from 112 families (See Table 1).

**Table 1:** A proposed typology of friendship discordance in MZ twins.

Discordance Category	Number of families described
Discordant peer victimisation	15
Discordant peer rejection	7
Fewer friends	39
Different friends	23
Different attitudes to friendship	23
Dependence on co-twin	5
**N**	**112**

Data for each of these categories were analysed separately. Before presenting the results of these analyses it is important to note that the data represent a series of case studies; although they can be used as the basis for testable hypotheses about peer relationships as an aspect of NSE, they do not in themselves speak to direction of effects. In this Results section all numbers in parentheses represent the number of families who reported a particular cause, correlate or consequence of the type of peer discordance being presented. Also, where diagnoses such as ADHD, eating disorders or social phobia are mentioned, they represent self-report data.

### Discordant peer victimisation

Twins were categorised as discordant for peer victimisation when they reported one twin being affected by the *actions* of others who deliberately and actively set out to hurt them. It can be differentiated from discordant peer rejection which was the code applied when one twin was affected by the *attitudes* of others, who may have ignored or disliked them. Fifteen twin pairs were categorised as discordant for peer victimisation.

Evidence of discordant peer victimisation in this sample included name-calling, cyberbullying and physical bullying which, in some cases, was persistent and very severe. One example of name-calling involved a twin who had been badly scarred by meningitis:

“He’s had to cope with the … nickname “Scar Boy”.”

In the most severe case of bullying the boy’s mother said:

“… he was beaten up most days on the bus, [they] punched his head against the windows, shouted abuse at him, chased him through the estate.”

Her bullied son added:

“…the police got involved because it became so bad. They’d jump me as I got off the bus, there’d be about 20 of them waiting for me.”

These fifteen families reported causes or sources of discordant bullying that included: discordance in sexuality [2]; behavioural disorders (e.g. ADHD, ASD) [3]; appearance (e.g. weight, skin problems) [5]; other relationships (e.g being liked by a bully’s girlfriend) [2]; or chance (e.g. being placed in a class with bullies) [6]. In general we did not include cases in which both twins experienced peer victimisation. However, we did include three cases in which both twins were bullied because participants reported either discordant causes or consequences of the reported victimisation. For example, in the case shared above, discordant responses to shared bullying led to worse attacks for one twin; this family reported how the fact that he stood up to the bullies (while his brother did not) led to violence escalating while the bullies left his co-twin alone.

In summary, in the current sample, MZ twins reported discordant experiences of peer victimisation that they perceived as being based on either chance occurrences or enhanced vulnerability (standing out in a way that others perceived as negative).

Participants reported the consequences of discordant peer victimisation as: discordance in confidence [6]; mental health (including eating disorders, self-harm, anxiety, suicide attempts, social phobia) [6]; future plans [4]; and social isolation [3]. In all cases the victimised twin reported worse outcomes. Alongside the negative outcomes there were three pairs in which a positive outcome was also acknowledged. This positive outcome was usually the result of escaping from the situation rather than of the bullying *per se*. For example, one bullied twin’s confidence improved when he left school for college. However, he still self-harmed and saw this as a result of being victimised at school. Perceived consequences of victimisation were very pronounced. In one case where the bullied twin had ADHD (which his mother explained with reference to twin-to-twin transfusion and perinatal experiences) she said:

He used to have marks on his arms and stuff from where he used to bite himself … He didn’t like himself very much.

Another mother, whose daughter had cut herself and taken an over-dose said:

Twin 2 is dissatisfied with herself and would like to reinvent herself somewhere else where her life would be more 'beautiful'.

While her mother attributed her difficulties to her personality as well as her peer problems her daughter said:

In my comprehensive school I had an unfortunate friendship which led to some bullying. This destroyed my confidence and relationships with other people … my anxiety, I feel, limits my career paths.

These data suggest that peer victimisation may have NSE effects on mental health, self-confidence, social isolation and future plans.

### Discordant peer rejection

Twins were coded as discordant for peer rejection when one twin experienced feeling left out, ignored or disliked by their peer group. This was evident in seven families. In one case the rejection was said to be imagined:

When Twin 2 was 3 years old she suffered severe hearing loss, eased by grommets. However, having had many months of not hearing, she didn't feel she had any friends as she never heard them when they were asking her to play. She changed from a wonderful, confident devil-may-care child to an introvert. She now has reduced hearing from scar tissue and her self-esteem has taken many years to recover—she is nearly there!

In most cases, however, family members agreed that one twin was in fact less accepted by their peer group. All presented theories for discordant acceptance of the twins. However, these causes were unsystematic and showed no clear pattern, all being mentioned in only one or two cases. Suggested causes included: discordant character judgement; sexuality; mental health problems (associated with school absence); protecting a vulnerable co-twin; and chance.

In terms of perceived consequences, again there was no systematic pattern except in the sense that outcomes tended to be more negative for the rejected twin. Suggested outcomes included: social isolation; reduced confidence *“[she] lost some of her sparkle”*; and changed future plans:

My twin doesn't want kids or anyone in her life, she just wants to move abroad.

As with victimisation, where outcomes were positive this was seen as the result of escaping the situation. One case, for example, involved gender dysphoria (a disorder in which individuals experience distress caused by a mismatch between their biological sex and their gender identity). The twin in question, who returned to school after the summer identifying as male and was subject to “snide comments”, said:

I think due to the discrimination I have faced since coming out in public and mainly school, I have become much more vulnerable and scared.

However, he also said that on going to university his confidence improved. As with victimisation the hypothetical causes of discordant peer rejection appear to be related to chance and enhanced vulnerability, and the consequences were generally negative and serious for the rejected twin. It may be possible to combine hypotheses related to peer victimisation and peer rejection.

### Fewer friends

Thirty-nine families reported one twin having fewer friends than the other. In a minority of cases [7] this was considered to be a positive situation in which each twin had a friendship group of a size and closeness that suited their personality and preferences. In all of these cases participants cited personality and preference as the cause of discordance in peer group size. However, in all other cases [32], having fewer friends was perceived as a negative experience. One girl, who had missed a lot of school because of mental health problems, said:

I'm probably going to end up with no friends because of the panic disorder. That's something I haven't said before. No friends, and a crap job makes for a grim future, doesn't it?

When offering explanations for why one twin had fewer friends than the other, most participants cited pre-existing behavioural or psychological discordance. For example, 22 families cited reasons related to discordant personality, confidence and self-esteem.

Even as a baby, Twin 1 was always much quieter and less secure—he never wandered off at playgroups. Twin 2 is more easy-going.

Seven families cited discordant physical or psychological health as the reason why one twin had fewer friends. Differences included Attention Deficit Disorder, anxiety, autism, epilepsy and scoliosis.

I have scoliosis (from birth) which means I'm less flexible and less agile. I had to miss about 3 months of school in Year 10 so I missed out on lots of school trips. It also means I'm not as good at sport because it hurts to run and jump a lot. My twin is really good at sports like lacrosse, which I wish I could be good at …. I feel like she has more friends and people prefer her.

A smaller number of families cited discordant interests [1] or appearance [2].

The environmental hypotheses for discordant size of friendship group included: chance events (e.g. having a best friend leave, being in a different class) [5]; falling out with a group of peers [1]; and having a boyfriend [5]. In all five cases where having a boyfriend was cited as the reason that one twin ended up with fewer friends, participants said that the twin with the boyfriend ended up being more socially isolated and, in one particularly difficult case, one twin required counselling when her boyfriend committed suicide.

As with peer victimisation and peer rejection, having fewer friends than a co-twin was generally viewed as a negative non-shared experience that was triggered by behavioural discordance much more often than by discordant experience. It is important to note, however, that behavioural discordance in MZ twins must have NSE roots.

Perceived consequences of having fewer friends that were cited by more than three participants were: reduced confidence [5]; future plans [8]; and social isolation [10].

I am ready to leave home and become more independent, something that Uni life will offer me. My twin is happy to be in the comfort of home and a local college.

I have a lot more confidence compared to my twin, she rarely answers questions in lessons and never goes out apart from school. She lacks self-confidence and never starts conversations with people at parties and social gatherings. Her friendship circle tends to change every few months and doesn't have a particularly close relationship with anyone apart from me.

These data suggest the hypothesis that being unpopular (or less popular than others) may have NSE effects on outcomes including social isolation, confidence and future plans. However, it is also important to note that some people prefer small, close friendship groups and the data do not suggest any negative outcomes of this. On the contrary, these young people were more likely to be described as confident, independent, more likely to value friends and less subject to peer pressure. Popularity was not a key issue in their cases.

### Different friends

In 23 families twins and/or parents stated that the twins had different friends, without adding that one had fewer friends or that one was rejected or victimised by peers. In 17 of these cases they said that the reason for the twins having different friendship groups was that, at some point in their education, they had been split up and were therefore exposed to different peer groups. In seven of these cases they were split up by choice because they actively wanted the opportunity to be treated as individuals. For example, in one family one twin:

was keen to gain a little more independence and possibly to make a wider circle of friends not shared with her sister.

In eight cases they were split up by chance, in that they were allocated to different classes or educational settings (e.g. a different boarding house). In the remaining two cases in which twins were said to have different friends as a result of being split up, the reason for the split was unspecified. In addition, two families mentioned discordant personality and confidence as a reason for having different friendship groups; one mentioned discordant interests; and a final family cited parental encouragement to be individuals.

In terms of consequences the most common discordance reported by participants as a perceived result of having different friends was discordance in personality and confidence [13]. In general, the twin who had been more successful in making friends who were a good fit for them, and with whom they could be themselves, were reported to be more confident and/or outgoing than their co-twin.

We have had different friendship groups which have encouraged different personalities … My friends and family say that my twin is more mature and I am 'crazier'. I am more self confident.

In another family in which one twin had missed a lot of school as a result of cardiac surgery and other health problems, her co-twin said:

Her health problems cause a lot of her stress, especially around friends as she missed a year of school due to it, whereas I continued going to school and gained greater independence and confidence socially.

In four cases families perceived discordant interests to be an outcome of different peer groups and, in a further five, discordance in future plans. For instance, one twin said:

A lot of it is down to our friend differences. The people we spend time with generally influence our behaviour somewhat. They have led to us finding our own separate interests.

Finally, in three families in which one twin had made friends who were a better fit for them, discordance in friendship quality and social life was reported as a perceived outcome of having different friends.

In summary, different friendship groups were primarily seen as the natural outcome of being split up and exposed to different peers. Non-shared peer groups were hypothesised to explain (a causal relationship) discordance in personality, confidence, interests and friendship quality. Exploring whether having different friends can explain variance in these outcomes using a quantitative design is indicated.

### Different attitudes to friendship

In 23 families participants described discordance in attitudes to friendship. These families’ responses were characterised by a specific focus on attitude to having and being a friend, rather than the actual make-up of the peer group. In some cases the twins shared a friendship group and in others they did not. Five different explanations for discordant attitudes to friendship were suggested. In 11 cases participants said that one twin was more willing to make an effort to socialise than the other:

My twin likes to go out more than me. We both have the same 'friend group' but sometimes if an opportunity to go out turns up then I might say no and my twin would normally say yes.

In eight cases families said that one twin was motivated by a greater need for peer approval. For example:

Twin 1 wants to be accepted and in with the cool crowd. Twin 2 [is] more inwardly confident, not so worried what people think of him.

Five families said that discordant attitudes to friendship were driven by discordant confidence (caused by earlier discordance in, for example, OCD and anorexia) and four by discordant personality. Finally, two families said that discordant attitudes to friendship were triggered by the twin relationship and, in particular, within-pair comparisons.

Discordant outcomes of these different attitudes were suggested by 16 of the 23 families and included: discordance in social life [6]; future plans [3]; study habits [3]; a preference for fewer, closer friends [3]; personality [1]; and stability of friendships [1]. It was interesting to note that in 18 of the 23 cases discordance in outcome was either not specified [5] or was neutral in content [13]. That is, neither twin was seen as having gained an advantage over the other by their attitude to friendship.

In the remaining five cases worse outcomes were described for one twin and were seen as the result of their attitude to friendship, or of the situation or behaviour that was seen as underpinning their attitude to friendship. In one case the less sociable twin decided not to go to university as he did not feel confident enough to leave home. In one, the more sociable twin lacked focus on his studies and in another the twin who needed more peer approval was less open to trying new things. One twin reported losing social confidence as a result of anorexia:

I think when I developed anorexia at 13 my confidence and social skills and health suffered, and has lead us to be different types of people. My twin is how I believe I would have been if I hadn't got anorexia.

These responses support the selection hypothesis in that families reported behavioural discordance as underpinning different attitudes to friendship. In most cases participants were relaxed about what they saw as the ensuing discordance, feeling, in general, that it simply reflected individual preferences. It was notable that the reported outcome discordance also appeared to be the result of behavioural selection.

### Dependence on co-twin

Five families described discordance in experience of peer relations in the sense that one twin was dependent on the other; that is, one twin made friends and the other just ‘tagged along’. In four cases this was seen as the result of discordance in personality (factors such as extraversion) and in one the result of chance. In the pair where chance was cited the twins had previously attended separate schools and when they came together one knew more people than the other. When the twin who was new to the school tried to ‘tag along’ with her sister this caused some friction. Other than this, all five families described the outcome of this discordance within the twin relationship as a concern about how the dependent twin would cope in Further or Higher Education when they would be split from their co-twin. Hypotheses from this aspect of discordant peer relationships are not applicable beyond twins.

## Discussion

A substantial minority (23%) of participants in this wide-ranging study spontaneously described and discussed discordance in friendships and peer relationships when asked about within MZ twin pair differences. Their responses suggested six categories of discordance of which four (peer victimisation, peer rejection, fewer friends and different friends) can be interpreted as environmental variables. The other two categories were different attitudes to friendship and dependence on a co-twin, and these are more easily interpreted as behavioural variables, albeit with non-shared roots and flowers. Together they suggest avenues for future research into experiences of friendship as components of the non-shared environment.

### Discordant peer victimisation and peer rejection

A recent MZ differences study identified being bullied as an NSE experience that was predictive of psychiatric dysfunction for environmental (NSE) reasons [33]. A minority of participating families (n = 22; 4.4% of the full sample) in the current study described situations in which one twin was exposed to bullying or rejection by their peers. It was clear from families’ descriptions that they saw this discordance as the result of either chance or enhanced vulnerability in one twin and that, either way, they saw the experience as being linked to negative outcomes. In the current sample the types of enhanced vulnerability described included: one twin being gay; coming to terms with gender dysphoria; and discordance in appearance. In these cases the more vulnerable twin was described as evoking more hostile or negative reactions from their peer group. This offers support to the selection hypothesis but as an evocative rather than an active process. Previous research has found antisocial adolescents to choose or shape antisocial peers. These case studies suggest that vulnerability can evoke negative treatment. These families perceived peer victimisation and rejection (which they saw as an outcome of chance or discordant vulnerability) as having a causal influence on self-confidence, future plans and social isolation. Their perceptions align well with Silberg et al.’s finding that being bullied exerts a negative environmental influence and we suggest that this may be true even if the bullying (or rejection) is partially explained by a genetically influenced phenotype (enhanced vulnerability). Knowing that a link is mediated by environment to a much greater extent than by genes has implications for intervention which could be relevant to clinical psychologists and educational practitioners. For instance, if a screening questionnaire could identify children and young people who feel isolated, or simply have fewer friends than they would like, then schools may be able intervene in a way that is beneficial for the young person and enhances non-cognitive, educationally-relevant traits. In addition families suggested a causal NSE relationship between peer victimisation and mental health difficulties, offering further support to Silberg et al’s findings [33]. In summary, the current data provide support for both the selection and the causal hypotheses of non-shared peer relationships and suggest that peer relationships can explain NSE variance in a range of outcomes. Testable hypotheses suggested by these case studies are:

1 Enhanced vulnerability can explain NSE variance in peer victimisation and peer rejection.
2 Peer victimisation and peer rejection can explain NSE variance in self-confidence, future plans and social isolation.
3 Peer victimisation can explain NSE variance in mental health.

It will be possible to test these hypotheses empirically, in a longitudinal design, in the context of the Twins’ Early Development Study (TEDS).

Our study and that of Silberg et al. [33] also raise the question of whether severity of experience is linked with severity of outcome (if a causal relationship can be identified). Our data do not suggest that one type of peer relationship discordance is likely to explain more NSE variance than another but that more serious peer problems may be more likely to explain variance in more serious outcomes (e.g. diagnosed mental health problems rather than undiagnosed self-confidence issues). This too can be explored in the longitudinal research proposed above.

### Fewer friends

In 32 of the 39 cases in which one twin was said to have fewer friends than the other it would be reasonable to suggest that discordant popularity was being described. It is important to note though that in the remaining seven cases the twin with fewer friends was seen as happy, and sometimes happier, than their co-twin. In these cases the twin with fewer friends felt that their peer group was a good fit for them. In the 32 cases in which one twin was reported as being more popular than the other the majority of families suggested discordance in factors variously described as personality, confidence and self-esteem as a cause. It would be interesting to explore the antecedents of this discordance as it must necessarily be explained by NSE factors. A further seven families cited health discordance–a type of enhanced vulnerability which, in some cases, was linked to prolonged absence from school. Chance and romantic relationships were also cited as reasons for discordant popularity. In this case we can see evidence for the selection hypothesis involving both active (more confident young people developed bigger friendship groups) and evocative processes (ill and often absent young people attracted fewer friends).

As with peer rejection, discordance in popularity was said to also have a causal role and, in fact, to lead to discordance in the same outcomes: self-confidence, social isolation and popularity. Popularity can therefore join peer victimisation and peer rejection in hypotheses 1 and 2. These variables were perceived by the families in this study as being the outcomes of discordant chance, behaviour and vulnerability, and the cause of discordance in outcomes.

### Different friends

In some families participants said that the twins had different friends to each other. While it is true that twins in the other categories also often had different friends, in those cases families specified that one had fewer friends or was bullied or rejected. The 23 families in this category only said that they had different friends, not that the relationships were unequal. The vast majority [17] said that they had been split up and exposed to different peers either by chance or by choice. The remaining families suggested discordance in confidence, personality, interests and parental encouragement to be individuals as the reason the twins had different friendship groups.

Families did describe perceived causal NSE effects of having different friends. In particular they described discordance in confidence. This tended to be the outcome of discordance in finding friends who were perceived as a good ‘fit’ with whom individuals felt they could be themselves. Other perceived consequences included discordance in interests and future plans. These data therefore suggest a testable hypothesis that:

4 Friendships can explain NSE variance in confidence, interests and future plans.

This hypothesis can also be investigated within TEDS, controlling for genetic and shared environmental effects.

### Different attitudes to friendship and dependence on co-twin

These observed categories of discordance were quite different to the others and appear to represent causes or correlates of different experiences of friendship rather than describing the experience *per se*. Because dependence on a co-twin is not a relevant experience for the non-twin population of adolescents this category is not discussed here.

The different attitudes to friendship cited by families included: discordance in effort to socialise; need for peer approval; confidence; personality; and reactions to the twin relationship. These attitudes were seen as being associated with social life, future plans and study habits. It was interesting to note though that in most cases families did not see one twin as disadvantaged by their experience. In only 5 of 16 cases were outcomes presented as worse for one twin than the other. In most cases families suggested that each twin had accessed peer experiences that they were comfortable with and that suited them as individuals. Social life and study habits could be added to hypothesis 4.

### Selection or causation?

These data suggest evidence for both the selection and causation hypotheses of peer relationships. MZ discordance in experience of peer relationships is necessarily caused by NSE effects. In this study we have seen hypotheses relating to factors such as: enhanced vulnerability (health, sexuality, appearance); personality or confidence; and chance. It is notable that selection appeared, in the current study, to be more often mediated by evocative than active processes, something that has arguably been overlooked in the field’s focus on antisocial behaviour and deviant peers.

Discordant peer relationships that favoured one twin over the other were perceived by twins and their parents as having a causal relationship with discordance in self-confidence, future plans, social isolation and mental health. If we can pin down the environmental influences on discordant peer relationships, and both identify and understand the environmental mechanisms underpinning relationships between peer problems and a range of outcomes, we will enhance our ability to intervene to support those who are disadvantaged by problematic relationships with their peers. Discordant peer relationships in which one twin was not advantaged over the other–relationships where the peer experience was seen as different in kind rather than in quality–were seen as explaining discordance in confidence, interests, future plans, social life and study habits. We therefore have grounds for continuing to consider both processes in genetically-informed studies of the peer relationship.

### Limitations

We took an inductive approach in the current study. In one sense this was a strength of the research as it allowed us to identify explanations that emerged spontaneously. However, it remains likely that we would have received different answers had we taken a more deductive approach and asked specific questions about peer relationships. For example, more pairs may have provided information about their friendships had we asked for it directly. They may also have been triggered to identify peer relationship discordance as part of a multi-faceted explanation for behavioural discordance if asked directly. Furthermore, this case study design can suggest hypotheses but cannot speak to direction of effects.

A further limitation, mentioned earlier, is that our sample was not representative of UK adolescents. Although this does not matter for within-pair comparisons it would strengthen our study if we could seek the spontaneous views of people not fully represented in the data we have gathered here. On this point it is a limitation that we discovered that TEDS families were less willing to provide open-response data than they are to provide the closed-response data that we more typically gather. This may have biased our sample and may be reflected, for instance, in the higher levels of g and SES observed in the current study (compared to TEDS data more generally). It is possible that this problem applies more to written than verbal responses and this is something we could explore in future qualitative work.

The genetically informed typology of peer relationships that emerged from these data does not contain anything very surprising in the sense that these aspects of peer relationships have been linked with life outcomes in non-genetic literature for many years [e.g. 17]. The novel contribution made here is that we present a basis for empirically testing their role as aspects of NSE experience, and for studying the environmental mediation of relationships between peer experiences and a range of outcomes. This will help us to understand the mechanisms of associations between peer relationships and outcomes, and will also help us to map the non-shared environment so that it begins to emerge as a set of named experiences rather than a non-specific proportion of variance. Furthermore, the current findings offer support to Silberg et al.’s empirical finding [33] that bullying appears to have a causal and truly environmental influence on mental illness. This matters because NSE influences are likely to be particularly susceptible to well-designed interventions.

Finally, the results of this study are merely descriptive and, to have any impact, need to be used as a basis for theory building about NSE, and taken forward to empirical testing. In particular, theory that links the severity of a peer problem with the severity of outcome (if prediction can be established and is environmentally mediated) may form a useful basis for future studies of the origins of mental health and wellbeing.

### Future research

Our next step will be to take some of the hypotheses generated by this study and test them using a quantitative design and a genetically-sensitive sample such as TEDS. There are two approaches that can be considered here. One is to focus on experience of friendship as a predictor of the range of outcomes identified in this hypothesis-generating study: self-confidence; future plans; social isolation; mental health; and interests. Another would be to focus on a particular outcome and explore the extent to which aspects of the friendship experience can explain NSE variance in this outcome. Future plans or self-confidence represent particularly interesting variables to study in this way as they were mentioned as outcomes of almost all categories of friendship discordance. Equally, studying the role of peer victimisation, rejection and unpopularity in explaining NSE variance in social isolation, confidence and mental health could be a fruitful and beneficial line of inquiry.

## Supporting information

S1 File MZ differences screening questionnaire (parent). (PDF)

S2 File MZ differences screening questionnaire (twin). (PDF)
