# Correction: Do MZ twins have discordant experiences of friendship? A qualitative hypothesis-generating MZ twin differences study

**DOI:** 10.1371/journal.pone.0186565

**Published:** 2017-10-11

**Authors:** Kathryn Asbury, Nicola Moran, Robert Plomin

The following information is missing from the Funding section: TEDS is supported by a programme grant to RP from the UK Medical Research Council (MR/M021475/1 and previously G0901245), with additional support from the US National Institutes of Health (AG046938) and the European Commission (602768; 295366).
