# Supplementary material for: Do MZ twins have discordant experiences of friendship? A qualitative hypothesis-generating MZ twin differences study
Source: PLoS One. 2017 Jul 20;12(7):e0180521. doi: 10.1371/journal.pone.0180521 (PMC5519028; doi:10.1371/journal.pone.0180521)
Supplement: S1 File — (PDF) [file pone.0180521.s001.pdf]

Family ID: <familyID>

Parent name: <parentforename> <parent surname>

Understanding and influencing pupils' choices as they prepare to leave school

Please give as much detail in your answers as you can manage – we really want to know what you think!

*For questions 1 – 4 tick **YES** or **NO**. If your answer is **YES** please give details in the spaces provided (and on additional sheets of paper if necessary). If **NO** please move on to the next question.*

1. Did <twin1forename> and <twin2forename> perform differently from each other in their GCSEs (or equivalent)?

Yes ☐ **No** ☐

(We are interested in overall differences. For example, perhaps one twin achieved much better grades or a lot more passes than the other; one twin failed to sit their examinations; one didn't meet the entry requirements for what they wanted to do next. Or any other differences – if you're not sure something is what we're after please jot it down.)

a. Please describe the differences.

____
____
____
____
____
____

b. How would you explain these differences?

____
____
____
____
____
____
____

c. What effect have these differences had on <twin1forename> and <twin2forename>'s future plans?

____
____
____
____
____
____

2. Did <twin1forename> and <twin2forename> get different grades (more than one grade apart) in GCSE English, Maths or Science?

ENGLISH Yes ☐ No☐
MATHS Yes ☐ No☐
SCIENCE Yes ☐ No☐

a. Please describe the differences in each subject for which you answered **YES**.

____
____
____
____
____
____
____

b. How do you explain these differences?

____
____
____
____
____
____
____
____

3. Did <twin1forename> and <twin2forename>do different things after completing Year 11?
Yes ☐ No ☐
(e.g. perhaps one went on to study for further qualifications while the other left school; one chose to start work; one became unemployed; one chose arts and the other sciences; both started further education but one dropped out; one chose a vocational path and the other an academic path; one went to live away from home while the other did not.)

a. What were the different choices that they made?

____
____
____
____
____
____
____
____

b. Can you tell us a little about how you would explain these different choices?

c. How have their different choices affected their future plans?

4. Do <twin1forename> and <twin2forename> have different hopes for the future?

Yes ☐ **No** ☐

(When answering this question please think about education, careers, relationships, possessions or any other plans or aspirations. We do not expect you to write about all of these areas of life, just those in which their hopes and plans seem MOST different.)

a. What do you think <twin1forename> and <twin2forename> each hope to achieve or do in the future?

b. Why do you think <twin1forename> and <twin2forename>have chosen to aim for different futures?

5. **What are the major differences (not already described) that you notice between <twin1forename> and <twin2forename>, and how do you explain these differences?**
(We are interested in ANY MAJOR differences e.g. personality; talent; ability; lifestyle; behaviour; relationships with family; circle of friends; self-confidence; motivation, mental and physical health … anything at all that strikes you as a MAJOR difference between them.)

a. Please describe the major difference(s) between <twin1forename> and <twin2forename>.

b. How do you explain the difference(s) you see between them?

c. What effect have these differences had on <twin1forename> and <twin2forename>'s future plans?

Thank you for sharing this information about your family. We really appreciate it. It will be used to find new ways of helping young people to fulfil their potential within the education system.
