# Supplementary material for: Do MZ twins have discordant experiences of friendship? A qualitative hypothesis-generating MZ twin differences study
Source: PLoS One. 2017 Jul 20;12(7):e0180521. doi: 10.1371/journal.pone.0180521 (PMC5519028; doi:10.1371/journal.pone.0180521)
Supplement: S2 File — (PDF) [file pone.0180521.s002.pdf]

Family ID: <twinID>

Twin name: <twinforename> <twin surname>

Understanding and influencing pupils' choices as they prepare to leave school

Please give as much detail in your answers as you can manage – we really want to know what you think!

For questions 1 – 4 tick ***YES*** *or* ***NO****. If your answer is* ***YES*** *please give details in the spaces provided (and on additional sheets of paper if necessary). If* ***NO*** *please move on to the next question.*

1. Did you and your twin perform differently from each other in your GCSEs (or equivalent)?

Yes ☐ **No** ☐

(We are interested in overall differences. For example, perhaps one of you achieved much better grades or a lot more passes than the other; one failed to sit your examinations; one didn't meet the entry requirements for what you wanted to do next. Or any other differences – if you're not sure something is what we're after please jot it down.)

a. What were the differences?

____

____

____

____

____

____

____

b. How would you explain these differences?

____

____

____

____

____

____

____

c. What effect have these differences had on your future plans?

____

____

____

____

____

____

____

2. Did you and your twin get different grades (more than one grade apart) in GCSE English, Maths or Science?

**ENGLISH**	**Yes ☐**	**No☐**
**MATHS**	**Yes ☐**	**No☐**
**SCIENCE**	**Yes ☐**	**No☐**

a. Please describe the differences in each subject for which you answered **YES.**

b. How do you explain these differences?

3. Did you and your twin do different things after completing Year 11

Yes ☐ **No ☐**

(e.g. perhaps one of you went on to study for further qualifications while the other left school; one chose to start work; one became unemployed; one chose arts and the other sciences; both started further education but one dropped out; one chose a vocational path and the other an academic path; one went to live away from home while the other did not.)

a. What were the different choices that you made?

b. Can you tell us a little about how you explain these different choices?

c. How have your different choices affected your future plans?

4. Do you and your twin have different hopes for the future?

Yes ☐ **No** ☐

(When answering this question please think about education, careers, relationships, possessions or any other plans or aspirations. We do not expect you to write about all of these areas of life, just those in which you feel your hopes and plans are MOST different.)

a. What do each of you hope to achieve or do in the future?

b. Why do you think you have each chosen to aim for different futures?

5. **What are the major differences (not already described) between you and your twin, and how do you explain these differences?**
(We are interested in ANY MAJOR differences e.g. personality; talent; ability; lifestyle; behaviour; relationships with family; circle of friends; self-confidence; motivation; mental and physical health … anything at all that strikes you as a MAJOR difference between you).

a. Please describe the major differences between you and your twin.

____

____

____

____

____

____

____

____

b. How do you explain these differences between you?

____

____

____

____

____

____

____

____

c. What effect have these differences had on your plans for the future?

____

____

____

____

____

____

____

____

Thank you for sharing this information. We really appreciate it. It will be used to find new ways of helping young people to fulfil their potential within the education system.
